# Programming of Dopaminergic Neurons by Early Exposure to Sex Hormones: Effects on Morphine-Induced Accumbens Dopamine Release, Reward, and Locomotor Behavior in Male and Female Rats

**DOI:** 10.3389/fphar.2019.00295

**Published:** 2019-03-26

**Authors:** Victoria B. Velásquez, Gabriel A. Zamorano, Jonathan Martínez-Pinto, Christian Bonansco, Pablo Jara, Gonzalo E. Torres, Georgina M. Renard, Ramón Sotomayor-Zárate

**Affiliations:** ^1^Laboratorio de Neuroquímica y Neurofarmacología, Valparaíso, Chile; ^2^Laboratorio de Neurofisiología, Instituto de Fisiología, Facultad de Ciencias, Universidad de Valparaíso, Valparaíso, Chile; ^3^Facultad de Ciencias de la Salud, Universidad Autónoma de Chile, Santiago, Chile; ^4^Department of Pharmacology and Therapeutics, College of Medicine, University of Florida, Gainesville, FL, United States; ^5^Centro de Investigación Biomédica y Aplicada (CIBAP), Escuela de Medicina, Facultad de Ciencias Médicas, Universidad de Santiago de Chile, Santiago, Chile

**Keywords:** dopamine, morphine, microdialysis, estradiol, testosterone, programming, nucleus accumbens

## Abstract

Neonatal programming with sex hormones produces long-term functional changes in various tissues, including the brain. Previously, we demonstrated a higher content of dopamine and an increase in potassium-induced dopamine release in the nucleus accumbens of adult rats exposed to estradiol valerate. On the other hand, sex hormones also affect the opioid system increasing the expression of the μ opioid receptor and β-endorphins. Here, we investigated if neonatal programming with sex hormones alters the response to morphine during adulthood in rats and predispose them to neurochemical, rewarding and behavioral activating effects. We examined the effects of neonatal exposure to a single dose of estradiol valerate or testosterone propionate on morphine-induced (5 mg/kg, i.v.) dopamine release in the nucleus accumbens and morphine-induced (3 mg/kg, s.c.) locomotor activity and conditioned place preference when these rats were adults. Our results showed a significant increase in morphine-induced dopamine release in the nucleus accumbens of rats that were exposed neonatally to estradiol compared with control rats. This effect was correlated with higher place preference and locomotor activity induced by morphine in adult rats neonatally exposed to estradiol valerate. However, the effect of morphine on dopamine release and behaviors was similar in rats treated with testosterone compared to control rats. Additionally, the expression of mu (μ) opioid receptor, dopamine receptor type 1 (D_1_) and dopamine receptor type 2 (D_2_) in the nucleus accumbens of adult rats was not different after treatment with sex hormones. Taken together, our results demonstrated an enhancement of pharmacological effects produced by morphine in rats neonatally programmed with estradiol valerate, suggesting that early exposure to sex hormones could represent a vulnerability factor in the development of addiction to opioid drugs such as morphine and heroin in adulthood.

## Introduction

Drug addiction is defined as a chronic brain disease characterized by compulsive use of the drug, loss of control in drug intake and the emergence of a negative emotional state that is exacerbated by abstinence. In this context, opioids are highly addictive drugs of abuse linked with potent positive (pleasure/gratification) and negative (anxiety/stress) reinforcing effects (Koob et al., 2014). Preclinical studies also indicate that sex plays a critical role in drug addiction. In this sense, it has been shown that female rats have higher cocaine choice than males and the ovariectomization of rats reduces the cocaine choice (Kerstetter et al., 2012). On the other hand, fluctuations in sex hormone levels during the estrous cycle may or may not affect the self-administration of drugs of abuse. For example, cocaine choice is not affected during estrous cycle (Kerstetter et al., 2012), while heroin choice (an opioid most commonly used as a recreational drug for its euphoric effects) is reduced during proestrus phase of estrous cycle (Lacy et al., 2016). In addition, clinical studies have been shown prevalence that the abuse for opioid analgesics and tranquilizers is higher in women than men (UNODC, 2012), women increase drug intake and develop dependence more quickly than men (Becker and Hu, 2008) and when levels of estrogens are high and progesterone is low (follicular phase) the rewarding effects of cocaine are greater than the effects observed during luteal phase (progesterone is high) (Sofuoglu et al., 1999). These sex differences are associated with a modulation of the reward system (mesolimbic pathway) by sex hormones (Cummings et al., 2014; Becker and Koob, 2016; Becker et al., 2016). The mesolimbic pathway is comprised of dopamine (DA) neurons from the ventral tegmental area (VTA) projecting primarily to the nucleus accumbens (NAcc). This circuit is activated by natural rewards (Pfaus et al., 1990; Bassareo and Di Chiara, 1997) or drugs of abuse such as morphine (Di Chiara and Imperato, 1988), through an increase in NAcc DA release. It is known that DA neurons express androgen, estrogen and progesterone receptors (Kuppers et al., 2001; Ravizza et al., 2003; Creutz and Kritzer, 2004), so sex differences observed in human and animals studies could be explained in part by the activation of these receptors by sex hormones and the concomitant regulation in the expression of key proteins involved in DA neurotransmission (for review see Sotomayor-Zarate et al., 2014; Becker and Koob, 2016). The search for factors that can explain sex differences in vulnerability to drugs of abuse has led to the study of sensitive periods of development where various organs are immature. Accordingly, fetal and neonatal stages are periods highly sensitive to deleterious stimuli that can reprogram the normal physiology of different organs such as the brain. Our group has shown that neonatal exposure to estradiol affects dopaminergic brain areas involved in prolactin release (tuberoinfundibular pathway) (Sotomayor-Zarate et al., 2011), movement (nigrostriatal pathway) (Cruz et al., 2014) and reward (mesolimbic pathway) (Bonansco et al., 2018) in adulthood. We observed that neonatal administration of estradiol valerate (EV) increased DA levels and tyrosine hydroxylase (TH) expression in midbrain dopamine neurons of adult rats (Cruz et al., 2014; Espinosa et al., 2016; Bonansco et al., 2018). The increase in DA content is functionally associated with greater NAcc DA release induced by chemical depolarization with K^+^ (Espinosa et al., 2016) or by electrical stimulation (Bonansco et al., 2018). However, it has not been evaluated whether neonatal exposure to estradiol favors greater reward effects induced by morphine in adulthood. The aim of the present study was to investigate the influence of neonatal administration of EV and testosterone propionate (TP) on morphine-induced NAcc DA release, conditioned place preference (CPP) and locomotor stimulation in adult male and female rats. In addition, in accordance with The National Institutes of Health (NIH) guidelines (McCullough et al., 2014; Miller et al., 2017), this study was focused in the effects of neonatal sex hormones administration in male and female rats. We postulate that neonatal EV and TP administration will increase morphine activating effects such as NAcc DA release, CPP and locomotor activity in adult male and female rats.

## Materials and Methods

### Animals

105 males and 97 females Sprague Dawley pups from 16 litters were considered for the experiments. However, we excluded 14 rats (9 males and 5 females) that showed obvious signs of stress such as weight loss, porphyric secretions, piloerection and aggression behaviors, between others. The analyses were performed using 188 rats (96 males and 92 females) that showed a good state of health and they were assigned to the following experimental groups: female control (*n* = 29), male control (*n* = 34), female EV (*n* = 34), male EV (*n* = 33), female TP (*n* = 29) and male TP (*n* = 29). All rats were kept under the same conditions of temperature (21 ± 2°C), 12-h light-dark cycle (lights on at 08:00 h), food and water *ad libitum*. All experimental procedures were approved by the Ethics and Biosafety Committee from the Universidad de Valparaíso, and the Institutional Animal Experimentation Ethics Board and the Science Council (FONDECYT) of Chile. Efforts were made to minimize the number of rats used and their suffering.

### Drugs and Reagents

EV, TP, sesame oil, DA standard, EDTA, and 1-octanesulfonic acid were purchased from Sigma-Aldrich, Inc. (St. Louis, MO, United States). Morphine hydrochloride was obtained from Laboratorio Sanderson S.A. (ISPCH N° F-10903/16, Santiago, Chile). All other reagents were of analytical and molecular grade.

### Experimental Procedure

Male and female pups were single-injected at postnatal day (PND) 1 with EV (0.1 mg/50 μL of sesame oil s.c.), TP (0.1 mg/50 μL of sesame oil s.c.) or vehicle (control group: 50 μL of sesame oil s.c.). The doses of EV and TP used were previously published and produce long-term effects in reproductive and non-reproductive tissues such as the brain (Sotomayor-Zarate et al., 2011; Cruz et al., 2014; Espinosa et al., 2016). All pups were raised with a nursing mother until weaning age (PND21). After weaning, rats were housed in standard cages by experimental group and sex under the aforementioned vivarium conditions until PND60 for male and PND60-62 for female rats. Control females were tested in the diestrus phase of their cycles because both the EV and TP exposed rats treated rats are constantly arrested in this stage of the estrous (by action of the neonatal administration of hormones) (Cruz et al., 2014). The stage of estrous cycle was daily recorded from PND40 to the end of the study (PND60-62). The estrous cycle was assessed by analyzing the relative proportion of leukocytes, epithelial cells, and cornified cells in daily vaginal lavages, which characteristically change during the various stages of the estrous cycle. All the experimental groups were randomly assigned for the following experimental protocols.

### Determination of NAcc DA Release

We used *in vivo* brain microdialysis in anesthetized rats following a general protocol previously published (Sotomayor-Zarate et al., 2013, 2015). Basal and morphine-induced DA extracellular levels were measured through high performance liquid chromatography (HPLC) coupled to electrochemical detection (ED). Rats used for microdialysis experiments were 9 (4 females and 5 males), 10 (5 females and 5 males), and 9 (4 females and 5 males) for control, EV and TP groups, respectively.

#### *In vivo* Brain Microdialysis

At PND60, rats were deeply anesthetized with isoflurane (5% in 0.6 L/min air flow) in an induction chamber and placed in a stereotaxic apparatus (model 68002, RWD Life Science Co. Ltd., Shenzhen, China) with a mask to maintain anesthesia for all the experiment (isoflurane 2% in 0.6 L/min air flow), using an animal anesthesia system (model 510, RWD Life Science Co. Ltd., Shenzhen, China). Body temperature was maintained at 37°C with an electrical blanket controlled by a thermostat. Concentric brain microdialysis probes (2 mm membrane length, model MAB 2.4.12, 35 kDa cut-off, Microbiotech AB, Stockholm, Sweden) were implanted in the NAcc using the coordinates from the Rat Brain Atlas (Paxinos and Watson, 2009) (NAcc: 1.50 mm posterior, 1.50 mm lateral, and 7.8 mm ventral to brain surface). Microdialysis probes were perfused with artificial cerebrospinal fluid (aCSF in mM: NaCl 147; KCl 2.7; CaCl_2_ 1.2; MgCl_2_ 0.85; pH 7.4) at a rate of 1 μL/min using an infusion pump (model RWD 210, RWD Life Science Co. Ltd., Shenzhen, China). After a stabilization period of 90 min, three perfusion samples were collected every 20 min in 3 μL of 0.2 M perchloric acid. At 60 min, an intravenous (i.v.) morphine injection (5 mg/kg) was administered to the rats. The dose of morphine has been previously published and injected by the same route of administration (Sotomayor et al., 2005). Seven perfusion samples were collected after morphine injection and all the perfusion samples were maintained on ice during the experiment and stored at -80°C until analysis. At the end of each experiment, rats were euthanized by decapitation with a guillotine (model 51330, Stoelting^TM^ Co., Wood Dale, IL, United States) and brains were quickly removed and stored in formalin. Brain sections of 50 μm were stained with Leishman’s eosin methylene blue solution (Cat. #1.05387.0500, Merck KGaA, Darmstadt, Germany) to verify probe location. Placement of the microdialysis probe was examined microscopically.

#### DA Quantification

Ten microliters of each dialysate sample were injected to the HPLC-ED system with the following equipment: An isocratic pump, (model PU-2080 Plus, Jasco Co. Ltd., Tokyo, Japan), a C18 column (model Kromasil 100-3.5-C18, AkzoNobel, Bohus, Sweden) and an electrochemical detector (set at 650 mV, 0.5 nA; model LC-4C, BAS, West Lafayette, IN, United States). The mobile phase, containing 0.1 M NaH_2_PO_4_, 1.0 mM 1-octanesulfonic acid, 1.0 mM EDTA and 8.0% (v/v) CH_3_CN (pH 3.4) was pumped at a flow rate of 125 μL/min. DA extracellular levels were assessed by comparing the respective peak area and elution time of the sample with a reference standard and the quantification was performed using a calibration curve for each neurotransmitter (Program ChromPass, Jasco Co. Ltd., Tokyo, Japan).

### Behavioral Studies

One hundred and sixty rats were used for CPP (*n* = 90) and locomotor activity (*n* = 70) experiments.

#### Conditioned Place Preference

Rats used for CPP were assigned to the following experimental groups: female control (*n* = 15), male control (*n* = 16), female EV (*n* = 16), male EV (*n* = 15), female TP (*n* = 14) and male TP (*n* = 14).

The CPP apparatus and the protocol used were previously described (Bonansco et al., 2018). Briefly, the conditioning protocol consisted of three parts: pre-test (one day before the conditioning period), the conditioning period and the test (24 h after the last injection). For the pre-test and the test, rats were placed in the neutral-gray center compartment with the two guillotine doors open and were allowed to explore the entire apparatus (two outer compartments) for 15 min. The time spent in each compartment was measured by analyzing the recordings obtained by internet protocol (IP) cameras (LX-C202 model; Lynx Security, China) fixed above each place preference apparatus and connected to a computer in another room. During the conditioning period (3 days) the non-preferred compartment (the white side) was associated with the reward induced by morphine (3 mg/kg s.c., once per day). The morphine dose and conditioning period used in this work it was previously published (Michaels and Holtzman, 2008).

The time difference (ΔT) in seconds between the time spent in the white compartment on the test day and the pre-test day was used to determine the grade of conditioning in the rats.

#### Locomotor Activity

Rats used for measure distance traveled were assigned to the following experimental groups: female control (*n* = 10), male control (*n* = 13), female EV (*n* = 13), male EV (*n* = 13), female TP (*n* = 11) and male TP (*n* = 10).

The protocol used for locomotor activity experiments was previously described (Cruz et al., 2014; Dib et al., 2018). Briefly, rats were habituated to the test room for 1 h before starting the experimental protocol. Basal and morphine-induced horizontal locomotor activity was measured using an acrylic box (22 × 44 × 28 cm). Basal locomotor activity was registered for 30 min and then rats were injected with physiological saline solution (1 mL/kg, s.c.) or morphine (3 mg/kg, s.c.), recording the locomotor activity for 180 min. The dose of morphine used in our behavioral experiments was chosen to produce selective locomotor hyperactivity (Neisewander and Bardo, 1987; Di Chiara and Imperato, 1988). Higher doses of morphine (between 10 and 30 mg/kg) produce sedation and thus, reduce locomotor activity (Magnus-Ellenbroek and Havemann-Reinecke, 1993; Spanagel et al., 1993). Each complete session of locomotor activity (210 min) was recorded with an IP camera (LX-C202 model; Lynx Security, China) and the total traveled distance (m) was analyzed using ANY-Maze software (Stoelting Co., Wood Dale, IL, United States). Test cages were wiped and cleaned with 5% ethanol solution between trials.

### qRT-PCR

Rats of the experimental groups injected with saline and used to measure locomotor activity were decapitated with a guillotine and the brain was removed. The NAcc was microdissected at 4°C using micro-punch, weighed on an analytical balance and stored at -80°C for further analysis. Real-time qRT-PCR was used to determine whether the mRNA encoding μ, D_1_ and D_2_ receptors changed in NAcc and VTA of adult female and male rats exposed to sex hormones at PND1. Total RNA was extracted using the E.Z.N.A.^®^ Total RNA Kit I (Cat. #R6834-02; Omega Biotek) according to the manufacturer’s instructions. RNA was quantified using the microplate Spectrophotometer Epoch (BioTek Inc., Winooski, VT, United States), and RNA integrity was assessed through agarose gel electrophoresis. Total RNA from each sample were reverse transcribed with PrimeScript RT reagent Kit (Cat. #RR047A; TaKaRa, Bio Inc., CA, United States), according to the manufacturer’s instructions. Real-time RT-PCR was performed using Taqman assays for the following genes purchased from Thermo Fisher Scientific: *Drd1* (Cat: Rn03062203_s1, FAM dye-labeled), *Drd2* (Cat: Rn00561126_m1, VIC dye-labeled) and *Oprm1* (Cat: Rn01430371_m1, FAM dye-labeled). The cycle conditions were 50°C for 2 min, 95°C for 2 min, 40 cycles for 95°C for 3 s and 60°C for 30 s. The relative quantification method was performed using Rn45s (Cat: Rn03928990_g1, VIC dye labeled) as an endogenous control. Results were expressed as fold change by the 2^-ΔΔCT^ method (Livak and Schmittgen, 2001).

### Statistical Analysis

Data were expressed as mean ± SEM. Two-way ANOVA followed by Newman–Keuls *post hoc* test analysis were performed for all experiments to determine a significant interaction of treatment by sex. The statistical analyses were carried out with GraphPad Prism v6.0 (GraphPad Software, San Diego, CA, United States) and *P* < 0.05 was considered statistically significant.

## Results

Our data show that neonatal exposure to EV increases the rewarding effects of morphine and it represents a vulnerability factor that may enhance the development of opioid dependence.

### Long-Lasting Effects of Neonatal Sex Hormones Administration on Morphine-Induced NAcc DA Release in Adult Rats

**Figure 1:**
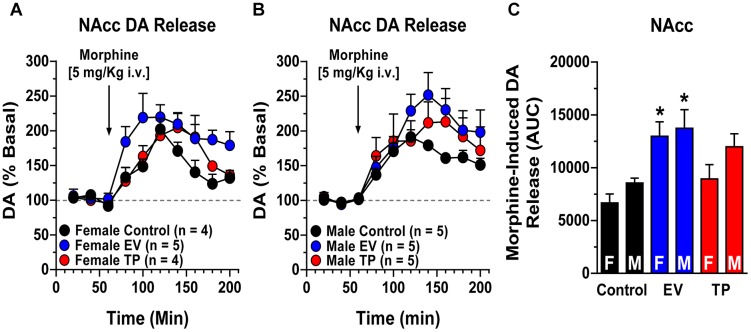
Morphine-induced NAcc DA release using *in vivo* brain microdialysis. Panels **A** and **B** Time course of NAcc DA release expressed as percentage of baseline (mean ± SEM) in female **(A)** and male **(B)** adult rats exposed to neonatal administration to estradiol valerate (EV) or testosterone propionate (TP). At postnatal day (PND) 60 microdialysis experiments were performed and after a stabilization period, three perfusion samples were collected. At 60 min a dose of morphine (5 mg/kg, i.v.) was injected. **(C)** shows an increase in the area under the curve (AUC) of morphine-induced NAcc DA release in female (F) and male (M) adult rats. ^∗^*P* < 0.05 for EV female rats vs. control female rats and for EV male rats vs. control male rats.

Figure 1A,B show time course of basal and morphine-induced extracellular levels of DA in NAcc. Figure 1C shows the area under the curve (AUC) that represents morphine-induced DA extracellular levels in control, EV and TP adult rats. In this context, neonatal exposure to EV in female and male rats increases morphine-induced NAcc DA release in the adulthood respect to control rats injected with vehicle (Two-way ANOVA followed by Newman–Keuls *post hoc* analysis; Interaction [*F*_(2,22)_ = 0.4001, *P* = 0.6751]; Sex [*F*_(1,22)_ = 3.328, *P* = 0.0817]; treatment [*F*_(2,22)_ = 10.17, *P* = 0.0007]).

### Long-Lasting Effects of Neonatal Sex Hormones Administration on Morphine-Induced CPP in Adult Rats

**Figure 2:**
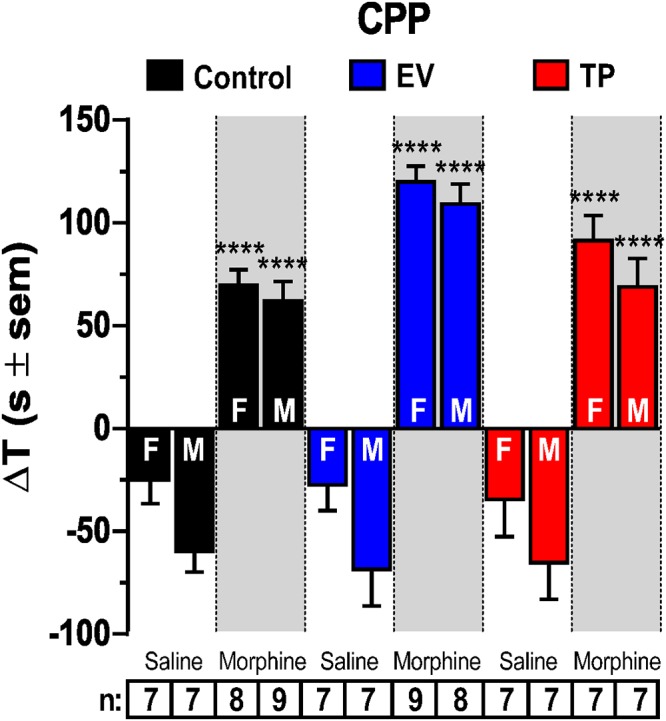
Conditioned place preference (CPP) to saline or morphine of adult female (F) and male (M) rats exposed to estradiol valerate (EV) and testosterone propionate (TP) at postnatal day (PND) 1. Data are shown as the time difference (ΔT) in seconds between the time spent in the white compartment in the test day and the pre-test day. Neonatal exposure to EV in female and male rats increases CPP induced by morphine (3 mg/kg, s.c.) respect neonatal injection of vehicle in female and male rats at PND 60 (^∗∗∗∗^*P* < 0.0001).

Figure 2 shows morphine-induced conditioned behavior in female and male adult rats exposed during the first 12 h of postnatal life to a single dose of EV or TP. As expected, morphine administration (3 mg/kg, s.c.) produced a significant increase in the time spent in the chamber paired to morphine injection vs. saline injection in female and male rats in all experimental groups (^∗∗∗∗^*P* < 0.0001). (Two-way ANOVA followed by Newman–Keuls *post hoc* analysis; Interaction [*F*_(5,78)_ = 0.5782, *P* = 0.7166]; Sex [*F*_(1,78)_ = 11.64, *P* = 0.0010]; treatment [*F*_(5,78)_ = 71.88, *P* < 0.0001]).

### Long-Lasting Effects of Neonatal Sex Hormones Administration on Morphine-Induced Locomotor Activity in Adult Rats

**Figure 3:**
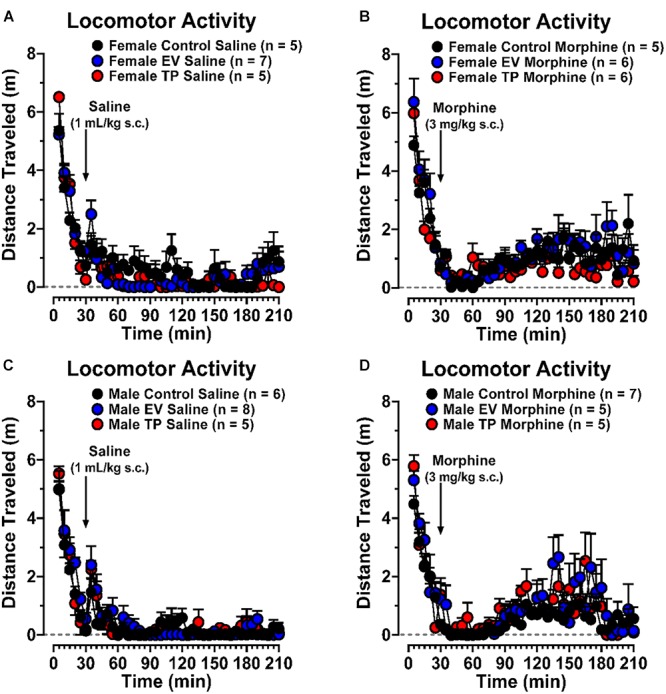
Time course of basal, saline-induced and morphine-induced locomotor activity in female **(A,B)** and male **(C,D)** control, EV and TP rats expressed in distance traveled (m).

**Figure 4:**
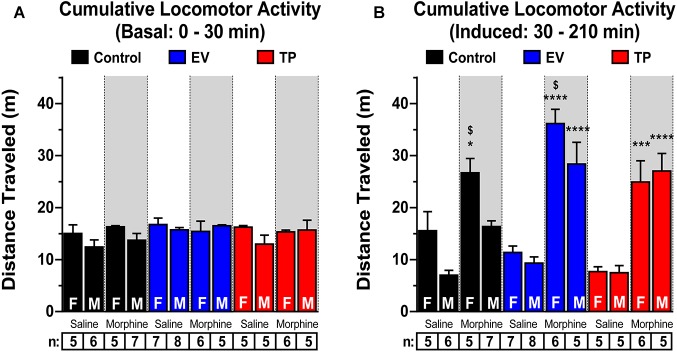
Morphine-induced locomotor activity in adult female (F) and male (M) rats exposed to estradiol valerate (EV) or testosterone propionate (TP) at PND 1. Cumulative locomotor activity from 0 to 30 min (basal) **(A)** and from 30 to 210 min (**B**, induced by saline or morphine injection) in female (F) and male (M) control, EV and TP rats ^∗^*P* < 0.05, ^∗∗∗^*P* < 0.001 and ^∗∗∗∗^*P* < 0.0001 versus saline female or saline male rats, respectively. ^$^*P* < 0.05 versus respective male rats.

Figure 3A,C show the temporal course of locomotor activity in the experimental groups that received only the saline (1 mL/kg, s.c.) injection. Morphine-induced locomotor activity was measured in adult female (Figure 3B) and male (Figure 3D) rats exposed during the first hours of postnatal life to EV or TP. Figure 4 shows basal (4A: 0–30 min) and post-injection (4B: 30–210 min) cumulative locomotor activities between experimental groups. Basal cumulative locomotor activity (Figure 4A) was not different in our experimental groups (Two-way ANOVA followed by Newman–Keuls *post hoc* analysis; Interaction [*F*_(5,58)_ = 1.012, *P* = 0.4192]; Sex [*F*_(1,58)_ = 3.424, *P* = 0.0694]; treatment [*F*_(5,58)_ = 1.102, *P* = 0.3695]). Figure 4B shows that cumulative locomotor activity induced by morphine (30 to 210 min) was significantly higher than cumulative locomotor activity produced by saline injection (^∗^*P* < 0.05, ^∗∗∗^*P* < 0.001, ^∗∗∗∗^*P* < 0.0001). On the other hand, control and EV female rats have a greater cumulative locomotor activity induced by morphine than control and EV male rats induced by morphine (^$^*P* < 0.05) (Two-way ANOVA followed by Newman–Keuls *post hoc* analysis; Interaction [*F*_(5,58)_ = 2.140, *P* = 0.0733]; Sex [*F*_(1,58)_ = 9.909, *P* = 0.0026]; treatment [*F*_(5,58)_ = 32.48, *P* < 0.0001]).

### Neonatal Exposure to Sex Hormones Does Not Affect the mRNA Expression of DA and μ Opioid Receptors in NAcc in Adulthood

**Figure 5:**
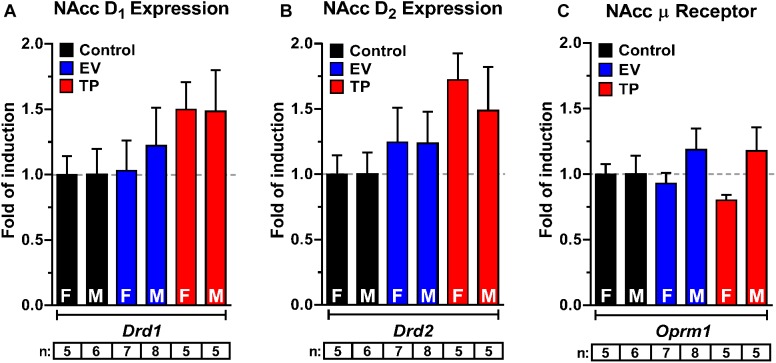
Neonatal exposure to estradiol valerate (EV) and testosterone propionate (TP) does not affect the expressions of *Drd1* **(A)**, *Drd2* **(B)**, and *Oprm1* **(C)** in nucleus accumbens (NAcc) of adult female (F) and male (M) rats. All data have been normalized for levels of 18S expression within the same sample. Results are expressed as fold of induction regard control group and represent the mean ± SEM.

mRNA expression for *Drd1* (Figure 5A), *Drd2* (Figure 5B), and μ opioid receptors (Figure 5C) were measured in NAcc of adult rats exposed during the first hours of post-natal life to a single dose of sex hormones. We did not observed a significant increase in *Drd1* (Two-way ANOVA followed by Newman–Keuls *post hoc* analysis; Interaction [*F*_(2,30)_ = 0.1006, *P* = 0.9046]; Sex [*F*_(1,30)_ = 0.08264, *P* = 0.7757]; treatment [*F*_(2,30)_ = 1.988, *P* = 0.1546]), *Drd2* (Two-way ANOVA followed by Newman–Keuls *post hoc* analysis; Interaction [*F*_(2,30)_ = 0.1482, *P* = 0.8629]; Sex [*F*_(1,30)_ = 0.1634, *P* = 0.6889]; treatment [*F*_(2,30)_ = 3.076, *P* = 0.0609]) and *Oprm1* (Two-way ANOVA followed by Newman–Keuls *post hoc* analysis; Interaction [*F*_(2,30)_ = 1.040, *P* = 0.3659]; Sex [*F*_(1,30)_ = 3.757, *P* = 0.0620]; treatment [*F*_(2,30)_ = 0.1506, *P* = 0.8608]), expressions in NAcc.

## Discussion

We have shown that neonatal EV administration produces a higher increase in morphine-induced NAcc DA release in both female and male adult rats compared to control rats. This effect correlates with a higher increase in morphine-induced CPP and locomotor activity in EV treated rats compared to controls. These findings indicate that neonatal exposure to sex hormones, especially estradiol, could reprogram the reward system and consequently alter to neurochemical, rewarding and behavioral activating effects of morphine in adult female and male rats.

### Neonatal Administration of Sex Hormones Increases Morphine-Induced NAcc DA Release in Adult Male and Female Rats

Morphine injection produces an increase in NAcc DA release in adult rats exposed during the first hours of postnatal life to EV compared to control and TP rats. Morphine is an opiate found naturally in the opium poppy plant and it is used therapeutically as a narcotic analgesic. One of the most important adverse effects of morphine is the highly addictive potential (similar to heroin). At the neurochemical level, morphine increases extracellular levels of DA in the NAcc (Di Chiara and Imperato, 1988; Tanda et al., 1997). In our experiments, neonatal EV exposure in rats produced an increase in NAcc DA release (Figure 1), CPP (Figure 2), and locomotor activity (Figure 3, 4) induced by morphine compared to control or TP-treated rats. In this context, several studies have shown that estradiol facilitates the motivation for drugs of abuse by interacting with reward system (Yoest et al., 2014). For example, estradiol increases cocaine self-administration, cocaine intake and motivation in ovariectomized rats (OVX) (Hu and Becker, 2008; Ramoa et al., 2013). On the other hand, locomotor activity induced by cocaine is lower in OVX than control (intact) rats (Walker et al., 2001). The neural and behavioral effects of estradiol on DA neurons have been associated with changes in expression levels of DA key proteins such as TH (rate-limiting enzyme of catecholamine biosynthesis), DAT (protein responsible for regulating DA levels through the uptake of DA) and DA receptors. In this sense, in OVX rats have been observed a reduction in levels of VTA TH and NAcc DAT that are restores after hormone replacement therapy (Chavez et al., 2010; Johnson et al., 2010). However, D_2_ levels are increased in NAcc and dorsolateral striatum (DLS) of OVX rats to compare controls rats and the estradiol replacement reduced D_2_ levels in NAcc and DLS of OVX rats (Chavez et al., 2010). The estradiol effects on these proteins are mediated by the activation of specific receptor such as estrogen receptor α (ERα) and estrogen receptor β (ERβ) expressed in reward system resulting in up- or down-regulation of gene expression (Simerly et al., 1990; Shughrue et al., 1997; Creutz and Kritzer, 2004). On the other hand, G protein-coupled estrogen receptor (GPER) produces rapid pharmacological response to estradiol (Gaudet et al., 2015). In NAcc and DLS the activation of GPER increases DA release induced by psychostimulant drugs in female rats (Becker, 1990; Cummings et al., 2014).

Regard our data, we previously demonstrated that neonatal exposure to EV during the first hours of postnatal life results in higher DA content in the mesolimbic pathway (VTA and NAcc) (Cruz et al., 2014; Espinosa et al., 2016) and increased NAcc DA release induced be a depolarizing stimulus (70 nM K^+^) (Espinosa et al., 2016; Bonansco et al., 2018). In addition, neonatal exposure to EV reprograms the electrophysiological properties of VTA DA neurons, favoring a higher firing frequency compared to control rats (Bonansco et al., 2018). All of these alterations in the dopaminergic system induced by neonatal EV administration may contribute to the increased morphine-induced NAcc DA release in EV rats.

Another possible mechanism involved in the increased morphine-induced NAcc DA release in adult rats exposed to EV during the first hours of life (Figure 1) might be a decrease in the inhibitory effect of GABAergic neurons on midbrain DA neurons. It has been shown that neonatal estradiol benzoate administration results in an enhanced PFC DA release in adult female rats and a decrease in brain levels of allopregnanolone (a potent neurosteroid that is a positive modulator of GABA_A_ receptors) when compared to control rats (Porcu et al., 2017). In addition, we showed that EV female adult rats have a decrease in GABA content in tuberal hypothalamus (Sotomayor-Zarate et al., 2011). Therefore, it is possible that this decrease in GABA content could also occur in the VTA, reducing the tonic inhibition of VTA DA neurons. Furthermore, the hyperpolarizing effect of morphine on VTA GABA neurons (Gysling and Wang, 1983; Johnson and North, 1992) in EV rats may favors a greater NAcc DA release than control rats. Interestingly, it has been shown that estradiol also stimulates VTA DA neurons through the inhibition of GABAergic afferents from medial preoptic area (anterior hypothalamus) to VTA, producing an increase in firing of DA neurons (Tobiansky et al., 2016).

### Neonatal Administration of Sex Hormones Increases Morphine-Induced CPP and Locomotor Activity in Adult Male and Female Rats

The repeated administration of morphine (3 mg/kg s.c.) results in conditioning in rats of both sexes. Interestingly, the degree of conditioning induced by morphine was higher in adult EV rats compared to control rats (Figure 2), reflecting the development of a more intense reward that could be associated to the increased NAcc DA release induced by morphine in EV rats (Figure 1). The neonatal exposure to EV not only produced an increase in CPP behavior associated to repeated morphine administration in adult rats (Figure 2), but also an increase in locomotor activity in response to an acute administration of morphine (see Figure 3). The dose of morphine used in this work was effective to induce locomotor activity when compared to saline injection in all experimental groups. Interestingly, although the basal locomotor activity (Figure 3) measured during first 30 min (in the absence of injection of saline or morphine) in all experimental groups were not different, we observed an increase in the locomotor activity induced by morphine regard to saline injection (30 to 210 min) (Figure 3).

In female EV rats the morphine-induced locomotor activity was higher compared to female control rats, while in the case of male rats, administration of both EV and TP resulted in an enhanced locomotor activity after acute morphine compared to control male rats (Figure 4). These neurochemical and behavioral results demonstrate that DA neurons programmed with sex hormones are susceptible to greater activation by opioids such as morphine. In this context, the effect observed in TP male rats on morphine-induced locomotor activity might result from partial aromatization of testosterone (T) to estradiol (E_2_) through cytochrome P450 aromatase, which is highly expressed in the midbrain (Raab et al., 1995). In previous work, we observed an increase in TH expression and DA levels in the substantia nigra and VTA in TP and EV male rats compared to controls. But, males treated with dihydrotestosterone (DHT), a non-aromatizable androgen at postnatal day 1 did not show changes in those parameters compared with controls, suggesting an estrogenic mechanism (Espinosa et al., 2016). On the other hand, published studies in intact female rats in diestrous phase of estrous cycle have a self-administration to drugs of abuse similar to males (Kerstetter et al., 2012; Lacy et al., 2016), in this work we observed that the neonatal administration of EV increases morphine-induced behaviors compared to control rats also in diestrous phase. Our results could suggest that the long-term effects observed on dopaminergic neurons by neonatal exposure to sex hormones could be due to permanent epigenetic changes. However, future studies would be needed.

Our results do not show changes in NAcc *Drd1* and *Drd2* expressions in female and male rats treated with EV and TP (Figure 5). In addition, we did not observe changes in NAcc μ opioid receptor expression, which suggests that the increase in the rewarding properties of morphine in EV rats might be due to an increase in DA synthesis (Bonansco et al., 2018) and DA release rather than changes in receptor expressions. However, the expression of proteins downstream of the activation of DA receptors should be explored, especially changes in the expression of the regulators of G-protein signaling (RGS) that are involved in locomotor responses to drugs of abuse (Gross et al., 2018) and modulated by estrogens (Silverman and Koenig, 2007).

## Conclusion

In conclusion, the alteration of sex hormones homeostasis during the early stages of development, either by a supra-physiological exposure to these hormones or by persistent exposure to endocrine disruptors (environmental pollutants) with estrogenic activity produce long-term effects not only in reproductive tissues, but also in non-reproductive tissues such as the brain. In this context, neonatal exposure to sex hormones can be considered a factor of vulnerability for substance use disorders such as morphine addiction and abuse. Finally, our experimental model of neonatal reprogramming allows exploration of the epigenetic mechanisms involved in DA neurons that may be responsible for the long-term effects observed in this work.

## Data Availability

All datasets generated for this study are included in the manuscript and/or the supplementary files.

## Author Contributions

VV performed microdialysis and conditioned place preference experiments. GZ performed the locomotor activity experiments. JM-P performed the qPCR experiments. CB, PJ, GT, GR, and RS-Z designed the experiments and interpreted the results. GR and RS-Z performed the statistical analysis of data and wrote the manuscript. RS-Z received funding for all experiments.

## Conflict of Interest Statement

The authors declare that the research was conducted in the absence of any commercial or financial relationships that could be construed as a potential conflict of interest.
